# 

**Published:** 1883-03

**Authors:** 


					﻿Contributors :
Alf. L. Looms, M.D................New York
Fessenden N. Otis, M.D.............
F. H. Bosworth, M.D................
J. L. Little, M.D..................
J. Williston Wright, M.D...........
C. E. Francis, D.D.S., M.D.S.......
A. J. C. Skene, M.D..........Brooklyn,	N. Y.
C. A. Marvin, D.D.S..........
Edwin T Darby, M.D., D.D.S.......Phila..	Pa.
P. S. Wales, M.D......Surg.-Gen’l	U. S. Navy
H. F. Campbell, M.D.............Augusta,	Ga.
C. H. Mastin, M.D., LL.D........Mobile,	Ala.
•J. J. Chisolm, M.D...........Baltimore,	Md.
C. F. Bevan, M.D..............
> I. E. Atkinson, M.D.......... "
i H. McGuire, M.D...............Richmond,	Va.
; F. P. Porcher, M.D.........Charleston,	S. C.
I Jas. T. Whittaker, M.D.......Cincinnati,	0.
J. Ransohoff, M.D., F.R.C.S....	“
F. Forchheimer, M.D................ "
A. R. Jackson, M.D..............Chicago.	Ill.
S. V. Clevenger, M.D.............
E. F. Ingals, M.D................
J. H. Carstens, M.D...........Detroit,	Mich.
PROSPECTUS
Fob Vol. IV., 1883, of the Independent
Pbactitionee.
This Jouenal is independent of colleges,
cliques, isms or any advertising firm; there-
fore impartiality pervades its columns in the
sole interest of the Profession.
It is cosmopolitan in its circulation and
scientific resources; consequently of equal
interest to the specialist and general prac-
titioner everywhere.
That which is new and useful in Medicine.
Surgery, Obstetrics, Dentistry, Pathology
and Popular Science is given to its readers
in an intelligent and concise style.
				

